# The Pancreatic Expression Database: 2018 update

**DOI:** 10.1093/nar/gkx955

**Published:** 2017-10-20

**Authors:** Jacek Marzec, Abu Z Dayem Ullah, Stefano Pirrò, Emanuela Gadaleta, Tatjana Crnogorac-Jurcevic, Nicholas R Lemoine, Hemant M Kocher, Claude Chelala

**Affiliations:** Bioinformatics Unit, Centre for Molecular Oncology, Barts Cancer Institute, Queen Mary University London, London EC1M 6BQ, UK; Centre for Molecular Oncology, Barts Cancer Institute, Queen Mary University London, London EC1M 6BQ, UK; Centre for Tumour Biology, Barts Cancer Institute, Queen Mary University London, London EC1M 6BQ, UK; Centre for Computational Biology, Life Sciences Initiative, Queen Mary University London, UK

## Abstract

The Pancreatic Expression Database (PED, http://www.pancreasexpression.org) continues to be a major resource for mining pancreatic –omics data a decade after its initial release. Here, we present recent updates to PED and describe its evolution into a comprehensive resource for extracting, analysing and integrating publicly available multi-omics datasets. A new analytical module has been implemented to run in parallel with the existing literature mining functions. This analytical module has been created using rich data content derived from pancreas-related specimens available through the major data repositories (GEO, ArrayExpress) and international initiatives (TCGA, GENIE, CCLE). Researchers have access to a host of functions to tailor analyses to meet their needs. Results are presented using interactive graphics that allow the molecular data to be visualized in a user-friendly manner. Furthermore, researchers are provided with the means to superimpose layers of molecular information to gain greater insight into alterations and the relationships between them. The literature-mining module has been improved with a redesigned web appearance, restructured query platforms and updated annotations. These updates to PED are in preparation for its integration with the Pancreatic Cancer Research Fund Tissue Bank (PCRFTB), a vital resource of pancreas cancer tissue for researchers to support and promote cutting-edge research.

## INTRODUCTION

Pancreatic Cancer (PC) is a death sentence for most of its patients and is projected to be one of the leading causes of cancer-related death by 2030, second only to lung cancer (1,2). Across the EU and US, an estimated 77% of PC patients will die within the first year of diagnosis and an estimated 94% of patients will die within 5 years (3,4). A multitude of studies investigated the pathogenesis of pancreatic malignancies and generated a huge volume of –omics data but these findings have not yet translated into clinical improvements. The Pancreatic Expression Database (PED) (5–7) was developed as a data repository to provide researchers with a single-entry point from which to manipulate, mine and integrate these heterogeneous and isolated findings into their own research. Although the emphasis is on pancreatic malignancies, PED also incorporates published findings on pancreatic precursor lesions, including pancreatic intraepithelial neoplasias (PanINs), intraductal papillary mucinous neoplasms (IPMNs) and mucinous cystic neoplasms (MCNs), as well as benign conditions such as chronic pancreatitis.

Since its inception in 2007, the literature mining module of PED has been enriched through manual selection of pancreas-related papers, followed by data curation and review of the reported findings. In this release, we also offer researchers the opportunity to explore experimental data *via* our web-based bioinformatics infrastructure.

Manual curation of the vast volume of experimental data available from public repositories is a time-consuming process. As such, PED uses an automated system for data selection and retrieval. This first-level data is made available immediately to the pancreatic cancer community for exploration.

Here, we present the recent release of PED, which has novel analytical modalities, capable of analyzing and integrating data generated using a range of technologies, and an enhanced data content and query-building process. With these additions and improvements, PED now offers an unprecedented opportunity to explore, analyze and integrate molecular data derived from a broad range of specimens from tissues and body fluids of healthy people or patients, cell lines and mouse models, thereby expanding its utility for pancreatic cancer research. In future, the established framework will facilitate the integration of PED with the Pancreatic Cancer Research Fund Tissue Bank (PCRFTB; https://www.thepancreastissuebank.org).

## NEW DEVELOPMENTS

### Analytical module

PED now contains a new module for the interactive analysis of a rich collection of publicly available multi-omics datasets in ArrayExpress, GEO, TCGA, GENIE and CCLE (8–12). It allows simple and efficient exploration of those datasets to perform user-specific queries that are, for example, not addressed or not directly discernible from the original publications.

#### Automated data selection and retrieval system

We have adopted the Smart Automatic Classification system (SMAC, *Pirrò *et al.*, in preparation*) to automate the selection and prioritization (13) of relevant articles accessed from PubMed. This is a marked departure from the previous releases of PED, where we relied on manual selection of articles and curation of reported high-level findings. The SMAC architecture has been expanded to identify any molecular data generated by the studies of interest. Where available, these associated experimental data files are downloaded from ArrayExpress or GEO and fed into the relevant analytical pipelines automatically. The automated system opens up the opportunity for periodic enrichment of our resource with minimal manual intervention.

#### Data content

**Figure 1.**
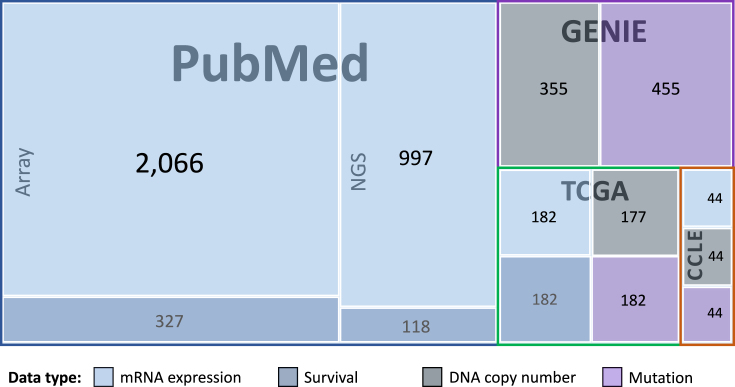
A Schematic overview of the data in the analytical module of PED.

The module is divided into four components, named after the core data sources: PubMed, TCGA, GENIE and CCLE. These hold expression, genomic and mutation profiles of pancreas-related samples or cell lines. Patient survival data is also available for a large number of patients in the PubMed and TCGA components (Figure 1).

#### Analytical features

A wide range of exploratory and investigative functions have been incorporated into PED (Table 1, see full details in the online user guide). The datasets extracted from different sources undergo a pre-processing workflow to ensure comparability and interoperability before being fed into the relevant analytical pipelines. For instance, the normalized mRNA expression data extracted from TCGA, CCLE and GEO/ArrayExpress undergo *z*-score transformation to ensure that the data from different studies are brought onto the same scale; the copy number data extracted from TCGA, GENIE and CCLE are binary-coded based on a hard threshold (2:Amplification; 1:Gain; -1:Loss; -2:Deletion).

**Table 1.** An overview of the features in the analytical module of PED

Data type	Analytical features	Unit of analysis	Component				Description	Display
			PubMed	TCGA	GENIE	CCLE		
Gene expression	Principal component analysis	Dataset	✓	✓		✓	Identification of key components of variability in the expression data	Scatter plot (2D, 3D)
								Scree plot
	Tumour purity	Dataset	✓	✓		✓	Estimate tumour purity and the presence of infiltrating stromal/immune cells for each sample	Scatter plot (3D) Table
	Expression profiles						Expression profile summarized across	
	- *gene-specific*	Gene	✓	✓		✓	- biological groups	Box plot
							- samples	Bar plot
	- *whole-genome*	Biological group	✓				Expression profile using median values of Z-transformed expression score for each gene across the genome	CIRCOS plot
	Correlation analysis	Multiple genes	✓	✓		✓	Pairwise correlations of genes presented by Pearson Product Moment Correlation Coefficients	Heatmap
	Gene networks	Gene(s)	✓	✓		✓	Interactions between genes of interest and their primary neighbours using human interactome dataset from MENTHA (14), overlaid with the expression data summarized across the groups	Network plot Table
Survival	Survival analysis	Gene	✓	✓			Univariate Cox proportional hazards regression analysis to assess the relationship between survival and gene expression	Kaplan-Meier plot
								
DNA copy number	Copy number analysis							
	- *gene-specific*	Multiple genes		✓	✓	✓	Genomic changes using DNA copy number of genes summarized across biological groups and samples	Heatmap
								
	- *whole-genome*	Biological group		✓	✓	✓	Genomic changes using the most frequent copy number alteration events for each gene across the genome	Frequency plot
Mutation	Mutations	Gene			✓		Frequency of different mutation types summarized across biological groups	Bar plot
Gene expression/DNA copy number/Mutation	Multi-omics data integration							
	- *gene-specific*	Gene		✓		✓	Integration of discrete genetic events, such as CNA events and mutations, or relative linear copy-number values with continuous mRNA abundance data	Box plot Scatter plot
	- *whole-genome*	Biological group		✓		✓	Integration of copy number alteration events with gene expression profile across the genome	CIRCOS plot

The exploratory features provide pre-computed results for principal component analysis (PCA), estimation of tumour purity and whole-genome view of expression or copy number alteration events. The investigative features include expression profiling, correlation analysis, survival analysis, copy number analysis, mutational profiling and gene network analysis, which are performed on the fly based on user-specific query. For TCGA and CCLE datasets that contain data generated from different technologies, an integrative modality is made available to investigate relationships between mutations, expression and copy number changes at single gene or whole-genome level.

#### Advanced visualizations

Most results are presented in an interactive and informative graphical format using the open source visualization library Plotly (15). The various statistical and scientific charts (see Table 1) allow users to visualize the annotation of data points, zoom to focus on area of interest, exclude/include subgroups in the data, and download as static image files of publication quality. The whole-genome view of expression and/or copy number data is generated using CIRCOS software (16), which also allows users to click on a particular chromosomal band to be redirected for a detailed view of the region of interest in the UCSC Genome Browser (17). Where applicable, results are also presented in an interactive tabular format with filtering, pagination, and sorting options, and are available for download in multiple formats.

### Improved literature mining module

The literature mining module has also been significantly updated to ensure that researchers are presented with a refined portal from which they can conduct intuitive biological queries. To this end, we have i) upgraded the BioMart data management system (18) to version 0.9 for improved query-optimization capability, and adopted MartExplorer GUI for intuitive query interface; ii) built a separate query mechanisms for beginners and advanced users. With the query interface containing fewer filters and attributes, beginners can now conduct quick and simple queries, whereas advanced users can access the original elaborate list of filters/attributes to conduct complex queries; iii) updated the annotations and mappings from Ensembl human gene annotations (19) release 63 to 90 to ensure up-to-date annotations with GRCh38/hg38 genome assembly.

### Updated documentation

We have incorporated a detailed user guide describing all the features and functionalities in PED. The user guide for the literature mining module has been updated to reflect all components of the new BioMart 0.9 query interface. An ‘Examples of use’ section is available, with practical demonstrations of how the literature mining module can be used for building biologically relevant queries. The user guide for the analytics module provides an overview of the types and sources of data, followed by an exhaustive description of the available analytical features.

## DISCUSSION AND FUTURE DIRECTIONS

Encouraged by the exponential growth of high-throughput multi-omics pancreas-related data, this release of PED has made the logical transition from a stand-alone data repository toward a cohesive research platform enriched with publicly available molecular data and equipped with the necessary analytical workflows required to conduct in-depth explorations and tailored investigations. Such a powerful combination of functionalities makes it possible to address challenging problems quickly, such as the discovery of non-invasive biomarkers or the identification of passenger deleted genes for cancer therapeutics. For instance, the concept of collateral lethality has recently been suggested as a potential therapeutic strategy for pancreatic cancer (20), focusing on the identification of co-deleted passenger genes neighbours. Using the literature mining module of PED, researchers can quickly search for homozygously co-deleted genes proximal to TSG, and their paralogous isoforms, a task that otherwise involves laborious data retrieval from a number of relevant studies and considerable additional data processing (see supplementary file 1). Furthermore, the analytics module of PED allows researchers to explore those genes in pancreas-related tissues and cancer cell lines, which aids in pinpointing potential candidates for further validation and pharmacological testing.

The updated infrastructure of PED is a major step toward its adoption as the bioinformatics platform of PCRFTB (21). PCRFTB aims to create a unique resource of biological materials and supportive clinical data from patients with different pancreatic malignancies or from healthy donors. PED will provide the bioinformatics platform to support cutting-edge translational research on these samples for the benefit of patients. In the future, both experimental data generated using samples obtained from the tissue bank and the corresponding published findings will be incorporated into PED. The seamless interoperability between the PCRFTB clinical data and PED modules will allow researchers to examine those findings and analyze the molecular data prior to applying for tissues, facilitating data sharing and reducing duplication of efforts.

## Supplementary Material

Supplementary Data
