# Supplementary material for: The Pancreatic Expression Database: 2018 update
Source: Nucleic Acids Res. 2017 Oct 20;46(Database issue):D1107–10. doi: 10.1093/nar/gkx955 (PMC5753364; doi:10.1093/nar/gkx955)
Supplement: Supplementary Data [file gkx955_supp.docx]

**SUPPLEMENTARY FILE 1**

**Example of use**

The design of PED and its query interface provides a powerful tool for the delivery of clinically relevant information. Here we present its application for searching homozygously co-deleted genes, proximal to deleted tumour suppressor genes (TSG), and their paralogs. Such collaterally deleted gene sets present pharmacologically targetable cancer-specific vulnerabilities for the development of personalised anti-cancer therapies - a novel concept named as “collateral lethality” (1). It has recently been reported as a prime therapeutic strategy for treatment of a substantial fraction of pancreatic cancer patients (2). The study has shown that the deletion of chromosome 18q21, observed in one-third of patients with pancreatic adenocarcinoma (PDAC), creates cancer-specific metabolic vulnerability upon targeting *ME3*. This occurs because the deletion of this region eliminates not only the TSG *SMAD4* but also neighbouring metabolic gene *ME2*, which is tolerated by cancer cells only because *ME2* has a functionally redundant paralog *ME3* located outside the deleted region. In this example, we demonstrate how PED literature mining and analytics module can be used to reproduce and validate those findings.

*Querying literature data with literature mining module*

This query involves identifying paralogs of genes reported as homozygously deleted in copy number studies. This can be done through the *Simple Query* interface for *Copy Number Alteration* data (Figure 1A). Upon selecting the *Copy Number Alteration* dataset, we expand the filter group and limit the query to PDAC samples (Figure 1B) and 200kb region around *SMAD4* (chr18: 51,028,394-51,085,045) (Figure 1C), and filter the data to include only regions with homozygous deletions (Figure 1D). We further refine the query to extract data reported by *Harada T et al.,* (Figure 1E) (3). For the Results, we select the “Target gene (reported)” and the copy number alteration “Subtype” (Figure 1F) from the attribute group for *Copy Number Alteration*. Finally, we establish the link between the genomics data and corresponding paralogous genes from the *Multi-omics* attribute group, which can be accessed by clicking on the arrow on the left side of the *Multi-omics* dataset title (Figure 1G). The query is submitted by clicking the “Results” button, followed by ordering the results table by “Target genes (reported)” column for better visualisation (Figure 1H). Two genes, *ME1* and *ME3*, are identified as paralogs of the *SMAD4* co-deleted metabolic gene *ME2*.

A


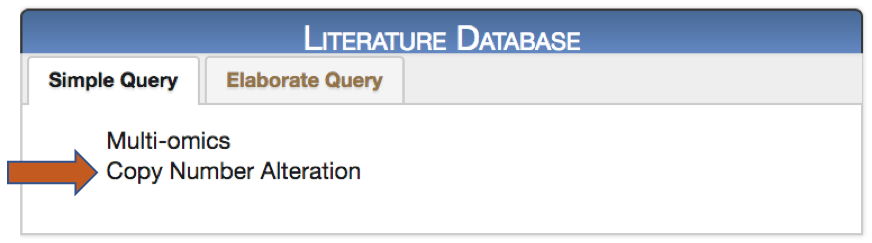


B
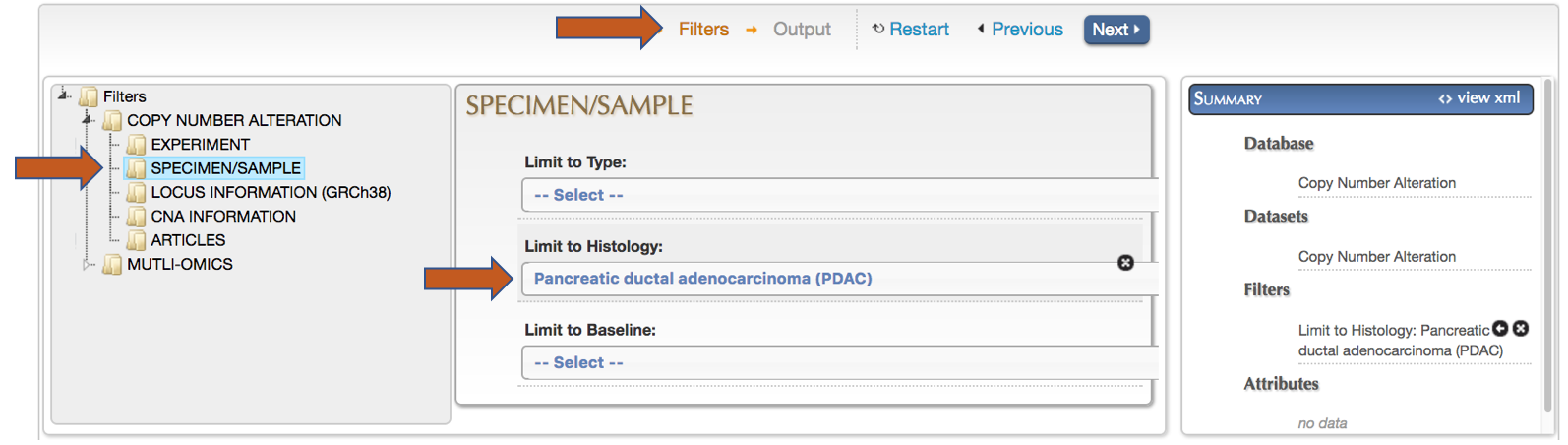


C


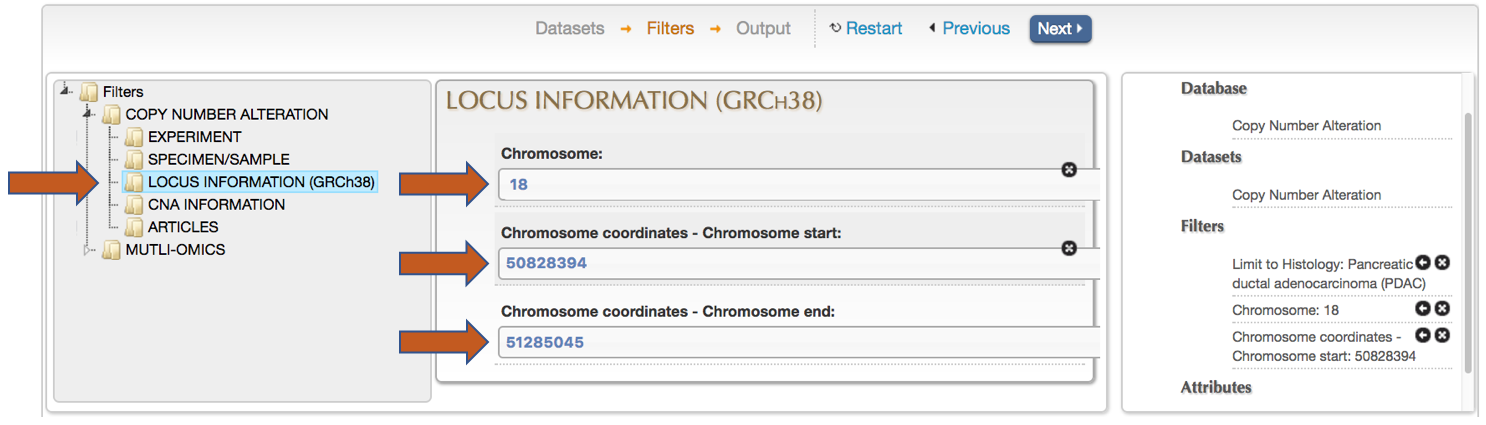


D


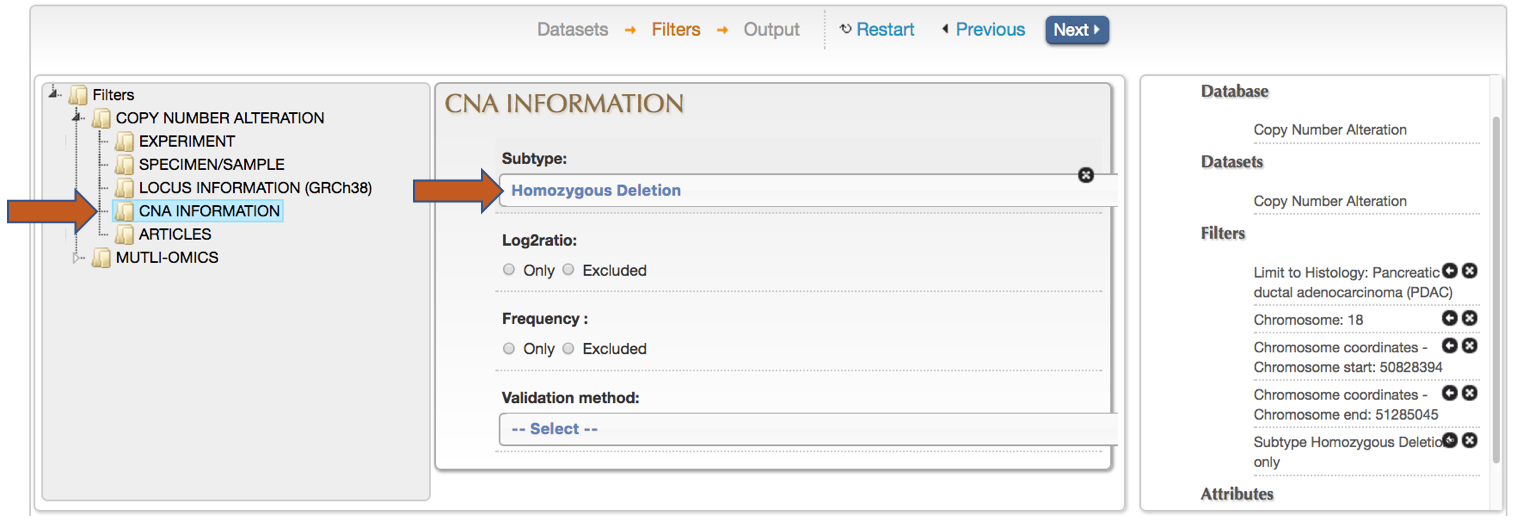


E


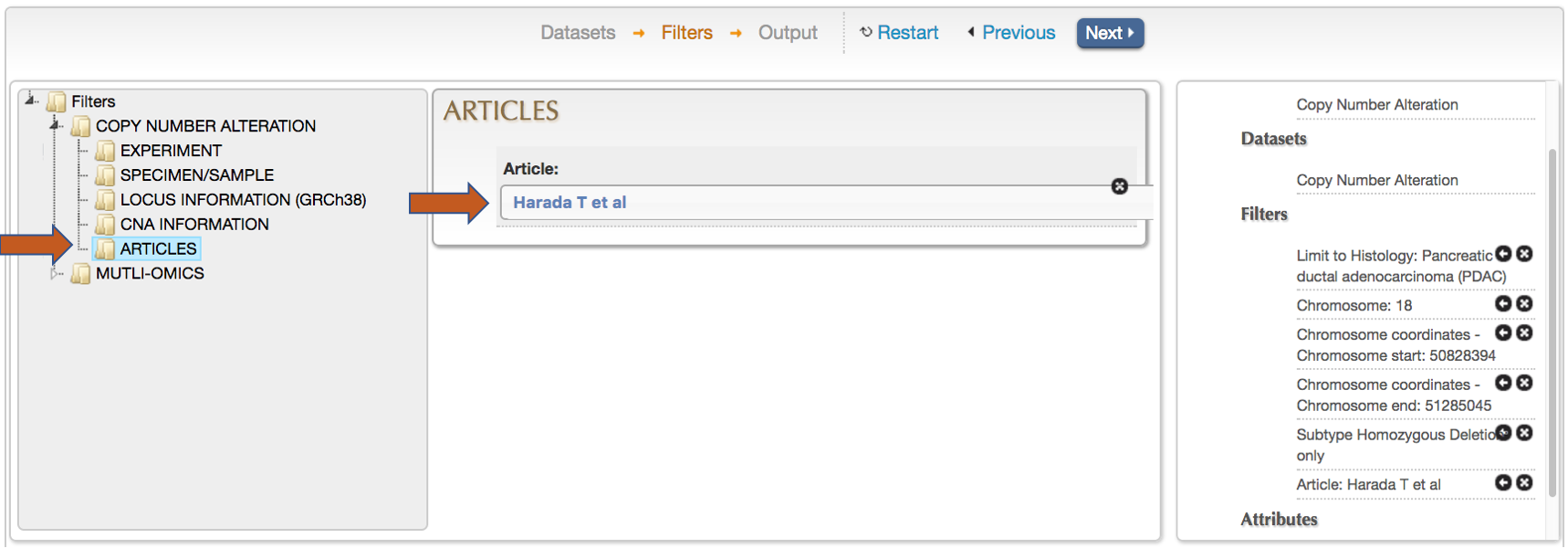


F


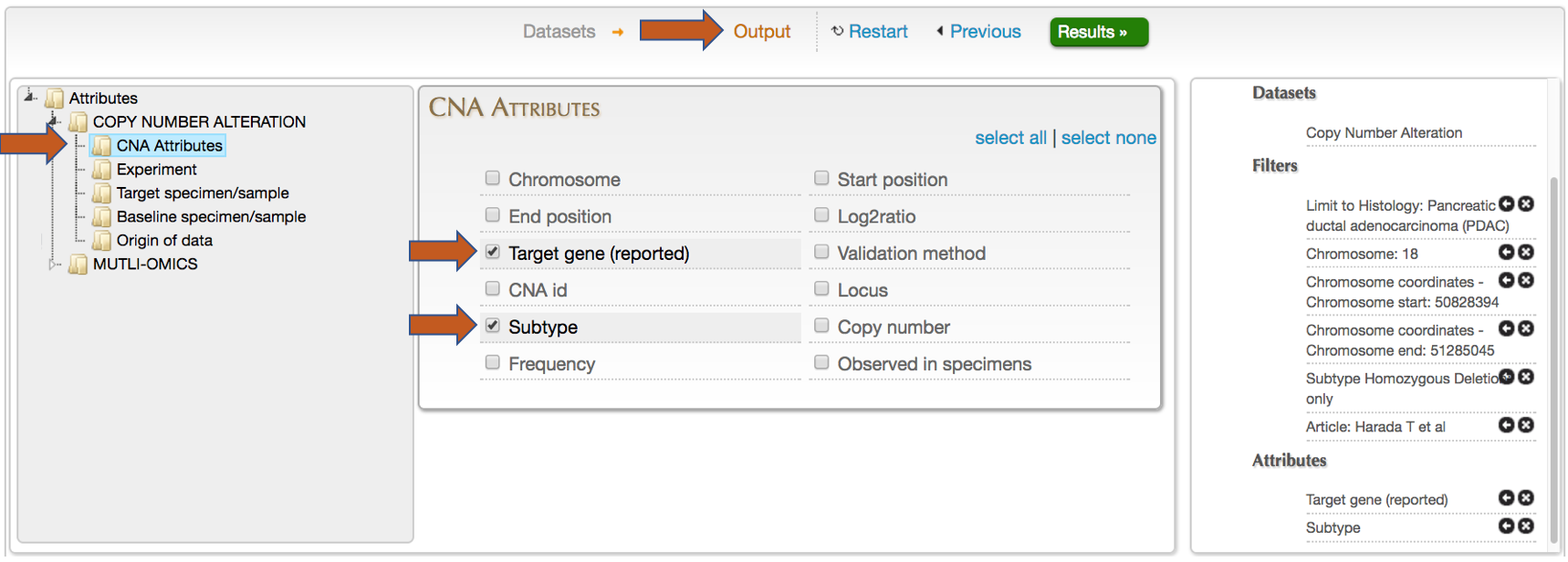


G


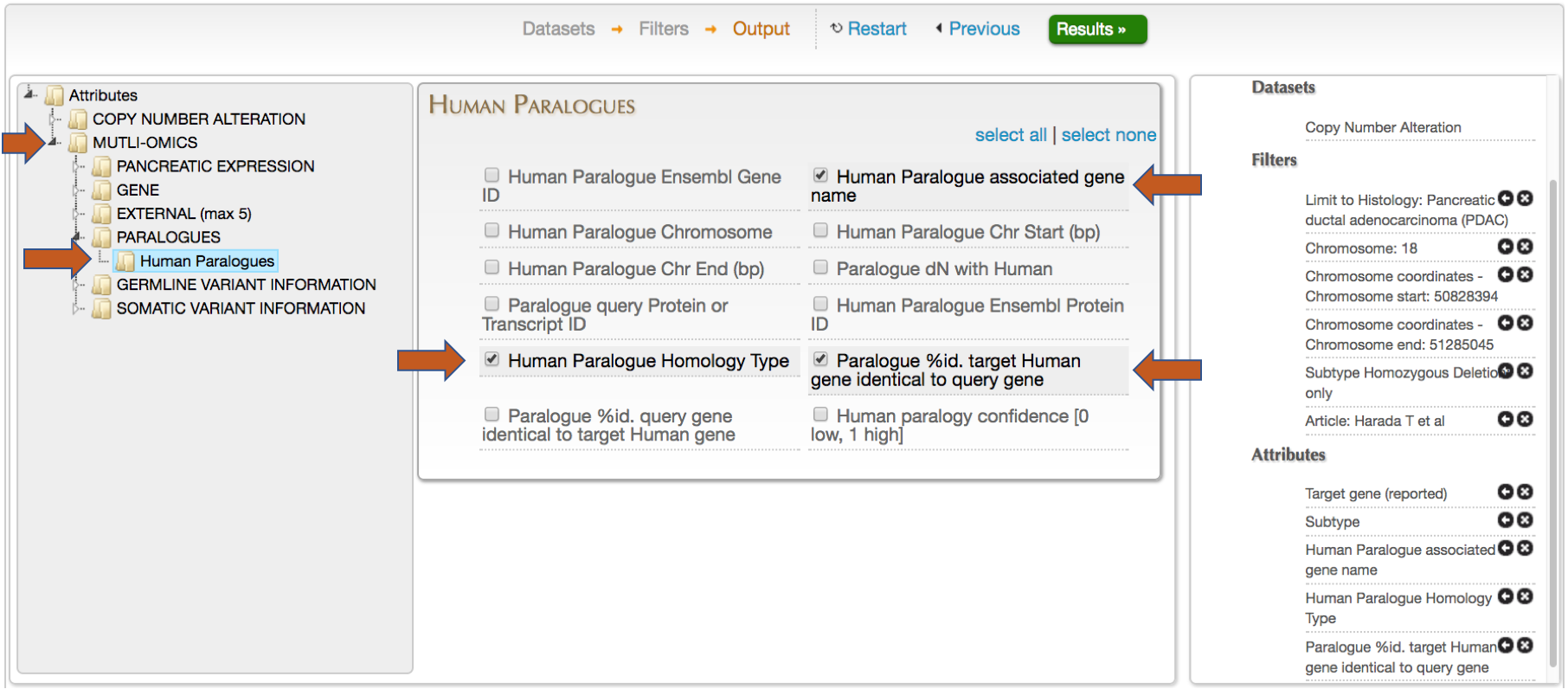


H


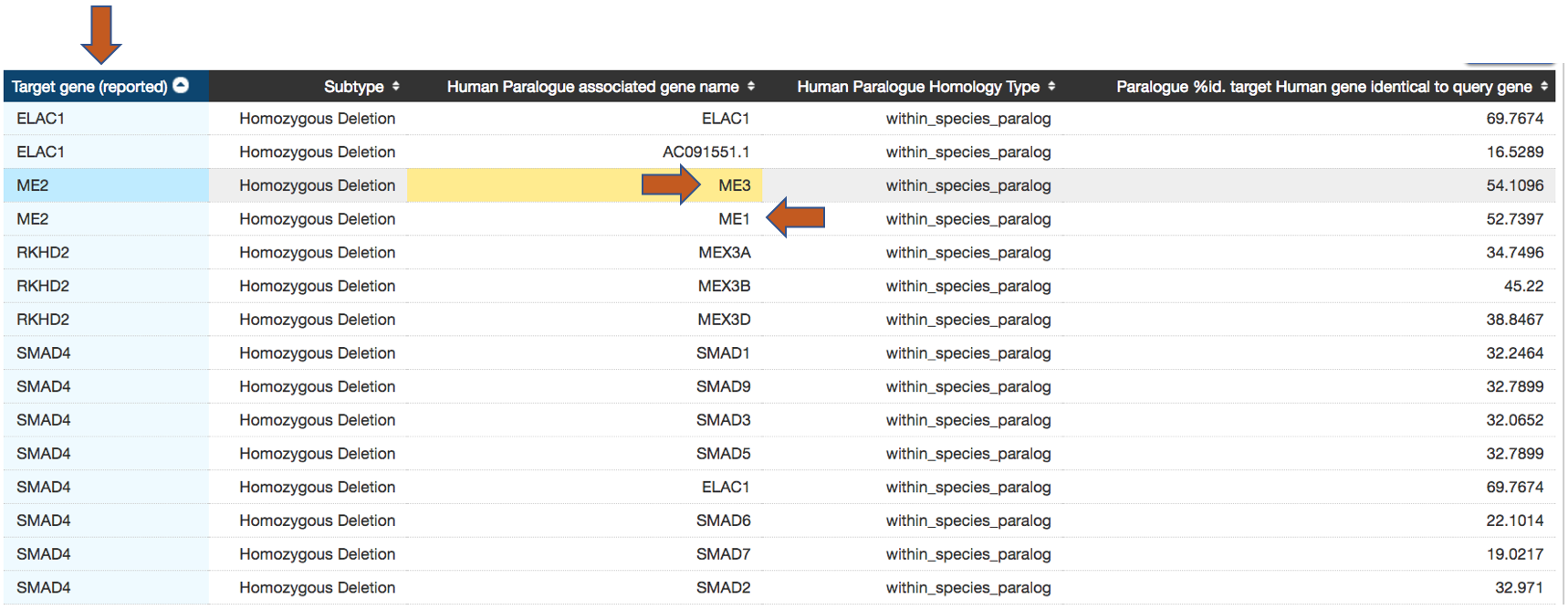


Figure 1. Querying literature data with PED literature mining module to search for *SMAD4* co-deleted genes and their paralogs.

*Exploration of molecular data with the analytical module*

The four genes within the deleted region 18q21, namely *SMAD4*, *ME2*, *ELAC1* and *MEX3C* (synonym for *RKHD2* gene), and the *ME2* paralogs, genes *ME1* and *ME3*, can be further explored at the molecular level using the PED analytics module. Using the TCGA pancreatic cancer cohort and cell lines from CCLE project, the expression, DNA copy number and mutation profiles of these genes can be examined.

By exploring the expression profiles of *ME2* (Figure 2A) and *ME3* (Figure 2B) paralogs in the TCGA cohort, one can clearly see the under-expression of *ME2* in the tumour samples, whereas the expression *ME3* is comparable between tumour and normal samples. The expression correlation analysis for the six genes in the TCGA cohort (Figure 2C) and across pancreatic cancer cell lines from CCLE (Figure 2D) show that the expression levels of *SMAD4* and *ME2*, *ELAC1* and *MEX3C* are highly correlated, indicating their co-deletion. Notably, in TCGA cohort, the expression of *ME3* demonstrate negative correlation when compared to *SMAD4* and *ME2*, which indicates its activation in the absence of the metabolic gene *ME2*. DNA copy number data across TCGA pancreatic cancer cohort (Figure 2E) and cell lines (Figure 2F) confirms co-deletion of *SMAD4* and *ME2* in majority of patients. Among the six genes, the loss or deletion of *ME3* are identified in the least number of patients/cell lines with some samples/cell lines demonstrating gain in that region, which may suggest activation of that gene. The integrative analysis using TCGA (Figure 2G,H) and CCLE (Figure 2I,J) datasets for *ME2* (Figure 2G,I) and *ME3* (Figure 2H,J) genes show fairly uncommon copy number alterations for *ME3*. However, loss/deletion of *ME2* are observed across many samples/cell lines with two TCGA patients presenting missense mutation, which supports *ME3* gene as a potential therapeutic target in *ME2*-deleted tumours. Finally, exploration of the expression profiles of *ME2* (Figure 2K) and *ME3* (Figure 2L) across primary pancreatic cancer cell lines (CCLE) show that two cell lines, Panc5.04 and PaTu8988S, present relative down-regulation of *ME2* and up-regulation of *ME3*, which confirms their potential to be used for validation and pharmacological testing.

A B

C D

E F

G H

I J

K L

Figure 2. Exploration of molecular data with PED Analytics.

**REFERENCES**

1. Muller,F.L., Aquilanti,E.A. and DePinho,R.A. (2015) Collateral Lethality: A new therapeutic strategy in oncology. *Trends Cancer*, **1**, 161–173.

2. Dey,P., Baddour,J., Muller,F., Wu,C.C., Wang,H., Liao,W.-T., Lan,Z., Chen,A., Gutschner,T., Kang,Y., *et al.* (2017) Genomic deletion of malic enzyme 2 confers collateral lethality in pancreatic cancer. *Nature*, **542**, 119–123.

3. Harada,T., Chelala,C., Bhakta,V., Chaplin,T., Caulee,K., Baril,P., Young,B.D. and Lemoine,N.R. (2008) Genome-wide DNA copy number analysis in pancreatic cancer using high-density single nucleotide polymorphism arrays. *Oncogene*, **27**, 1951–1960.
